# Berberine Hydrochloride Reduces the Intracellular Survival of *Salmonella* Typhimurium by Enhancing Host Autophagic Flux Through the Inhibition of the Type III Secretion System

**DOI:** 10.3390/biom15111589

**Published:** 2025-11-13

**Authors:** Jianan Huang, Jiaxing Lu, Conghui Wu, Sidi Chen, Tianyuan Chang, Lei Xu, Xihui Shen, Qadir Bakhsh, Baofu Qin, Weidong Qian, Yao Wang

**Affiliations:** 1 Shaanxi Key Laboratory of Agricultural and Environmental Microbiology, College of Life Sciences, Northwest A&F University, Yangling 712100, China; 2 Institute of Plant Sciences, University of Sindh, Jamshoro 76080, Sindh, Pakistan; 3 School of Food and Biological Engineering, Shaanxi University of Science and Technology, Xi’an 710021, China

**Keywords:** *Salmonella* Typhimurium, berberine hydrochloride (BH), type III secretion system (T3SS), autophagic flux

## Abstract

*Salmonella* Typhimurium, a significant intracellular foodborne pathogen, regulates host cell autophagy to achieve its own survival by injecting effector proteins into host cells via its type III secretion system (T3SS). Berberine hydrochloride (BH), an isoquinoline alkaloid derived from medicinal plants such as *Coptis chinensis*, has demonstrated potential antibacterial and immunomodulatory properties. However, the mechanisms by which BH combats *S.* Typhimurium by enhancing host autophagic flux through the inhibition of the type III secretion system remain to be fully elucidated. Here, we found that BH disrupts biofilm formation of *S.* Typhimurium, significantly inhibits the expression of genes associated with T3SS, and robustly enhances autophagy activity in macrophages infected with the pathogen. In a mouse model (C57BL/6 female 20 ± 1 g/mouse), BH significantly improved survival rates, reduced bacterial loads in tissues, and alleviated pathological damage. Molecular docking studies revealed that BH binds to key T3SS proteins, including SipB, SseA, and SsrB. These findings indicate that BH holds promise as a potentially effective therapeutic strategy for combating *S.* Typhimurium infections.

## 1. Introduction

*Salmonella* Typhimurium (*S.* Typhimurium), a major foodborne pathogen that causes millions of cases of gastroenteritis and invasive infections worldwide, promotes intracellular survival by mainly injecting effector proteins into host cells through the type III secretion system (T3SS) [1,2,3,4,5,6]. Inhibiting host autophagy through the T3SS to block intracellular pathogen clearance is also a key immune evasion strategy for this bacterium [7,8]. Given the increasing resistance of *Salmonella* to first-line antibiotics such as aminoglycosides and fluoroquinolones, dual-targeted therapy targeting virulence pathways (such as T3SS) and host defense mechanisms has become an urgent need to combat infections [9,10].

The natural product berberine hydrochloride (BH), known for its broad-spectrum antibacterial activity and immunomodulatory properties, has become a potential drug candidate by disrupting bacterial membrane structure, suppressing biofilm formation, and regulating inflammation [11,12,13,14,15]. BH, as an isoquinoline alkaloid extracted from medicinal plants such as *Coptis chinensis* Franch., has been widely studied for its broad-spectrum antibacterial activity against Gram-negative and Gram-positive pathogens [13,16]. In addition, BH also possesses unique pharmacokinetic characteristics that support its therapeutic potential—in rodent models, it is rapidly absorbed in the intestinal tract (reaching peak plasma concentration within 1–2 h), primarily metabolized in the liver via glucuronidation, while its half-life (1–2 h) may be extended through formulation adjustments [17,18]. Clinical studies have shown that BH exerts a potent inhibitory effect on *Salmonella* Typhimurium, primarily by disrupting biofilm formation and interfering with quorum sensing [19,20]. In addition to *Salmonella*, BH’s broad-spectrum activity against various pathogenic bacteria demonstrates its multi-target advantage [13,21,22]. Transcriptome analysis further showed that BH could downregulate genes associated with bacterial motility and stress response while regulating host immune response by inhibiting the production of pro-inflammatory cytokines [20,23].

Autophagy is a key defense mechanism for hosts against intracellular pathogens by forming autophagosomes with bilayer membrane to encapsulate bacteria [24,25]. By regulating the SIRT1/AMPK/mTOR signaling axis, autophagosomes fuse with lysosomes to degrade pathogens, thereby eliminating intracellular bacteria [7,26,27,28]. As a core hub of bacterial pathogenesis, T3SS uses effector proteins to reduce AMPK synthesis, thereby weakening its inhibitory effect on mTOR, further reducing the formation of ULK complexes, ultimately leading to a decrease in lysosomal synthesis, inhibiting autophagic clearance, and aiding bacterial immune evasion [7,29].

Existing studies have shown that BH significantly activates cellular autophagy by activating the AMPK pathway and inhibiting the mTOR signaling [30,31]. However, the mechanisms by which BH reduces the survival of *S.* Typhimurium by enhancing host autophagic flux through the inhibition of T3SS have yet to be fully elucidated. This study elucidates for the first time the mechanism by which BH regulates host autophagy via T3SS and establishes a novel therapeutic paradigm of “bacterial virulence inhibition and host defense enhancement” for *Salmonella*-induced gastroenteritis. These findings highlight the potential of BH in developing anti-infective drugs against *S.* Typhimurium.

## 2. Materials and Methods

### 2.1. Reagents

BH, with a purity of 99% or higher, was obtained from Chengdu Must Bio-Technology Co., Ltd., Chengdu, China. Dyes such as SYTO9 and propidium iodide (PI) were purchased from Invitrogen, a part of Thermo Fisher Scientific, Waltham, MA, USA.

### 2.2. Strains, Cells and Mice

The standard strain of *Salmonella* Typhimurium 14028 was purchased from Qingdao Haibo Biotechnology Co., Ltd., Qingdao, China, and was cultured in LB medium (Sangon Biotech, Shanghai, China) at 37 °C with shaking at 250 rpm. The mouse macrophage cell line Raw264.7 (American Type Culture Collection (ATCC), Manassas, VA, USA) was stored in our laboratory and cultured in DMEM medium supplemented with 10% fetal bovine serum (FBS) and 1% penicillin streptomycin. Six-to eight-week-old specific pathogen-free (SPF) grade C57BL/6 female mice (20 ± 1 g/mouse) were purchased from the Animal Center of Xi’an Jiaotong University.

### 2.3. Minimum Inhibitory Concentration (MIC) and Minimum Bactericidal Concentration (MBC)

The MIC of BH to 14028 strain was determined according to the Clinical and Laboratory Standards Institute (CLSI) M27-A2 protocol [32]. Fresh bacterial cultures were diluted in LB broth medium (Sangon Biotech, Shanghai, China) to an optical density of 0.5 at 600 nm (OD_600_), and 100 μL of the suspension was added to 96-well plates. Then, serial dilution was performed on BH solutions (prepared in LB medium) to achieve a final volume of 200 μL per well. Bacteria cultured in LB medium were used as negative controls, while bacteria cultured in LB medium containing 50 μg/mL of kanamycin were used as positive controls. Plates were incubated at 37 °C for 24 h, and OD_600_ was measured using a microplate reader (Thermo Fisher, Joensuu, Finland). To determine the MBC, 10 μL of culture from wells with no bacterial growth was inoculated onto LB agar plates and incubated at 37 °C for 18–24 h; the MBC was defined as the lowest BH concentration resulting in ≥99.9% reduction in viable colonies compared to the untreated control [33].

### 2.4. Growth Curve

Activated bacteria (in the exponential phase) were diluted in LB medium to an initial inoculation concentration of 1 × 10^5^ colony-forming units (CFU)/mL. The diluted suspensions were transferred to each well of a 24-well plate, establishing a wild-type (WT) control group and drug-treated groups. For drug-treated groups, BH was added to each well to achieve final concentrations ranging from the MIC to 1/8MIC. The culture plates were incubated at 37 °C, 200 μL was taken from each culture well every 2 h, and OD_600_ was measured to plot the bacterial growth curves [34].

### 2.5. Observation of Biofilm Formation

Bacterial culture and berberine hydrochloride solution were added to a 24-well plate pre-placed with glass coverslips, with final concentrations of 0 MIC (WT control), 1/2 MIC, and MIC. Plate was incubated at 37 °C with 50 rpm shaking for 24 h [35]. After washing the cell coverslips three times with PBS, biofilm analysis was performed as follows: (1) crystal violet staining for optical microscopy observation [36]; (2) sample preparation following the bacterial morphology protocol for FESEM analysis of biofilm architecture; (3) incubation of biofilms on coverslips with SYTO9/PI staining solution at room temperature in the dark for 20 min, followed by two PBS washes and visualization using CLSM.

### 2.6. Swimming Motility Assay

BH was added to 14028 strain cultures at an OD_600_ of 0.5 to final concentrations of MIC, 1/2MIC, and 0MIC (WT control), followed by incubation at 37 °C with shaking at 200 rpm for 12 h. Cultures were collected, diluted to an OD_600_ of 0.8 (logarithmic growth phase), and then 2 μL of the suspension was spotted at the center of culture dishes containing 0.4% semi-solid motility medium (allowing the inoculum to penetrate the medium surface without reaching the plate bottom). The formula for the motility medium is as follows: dissolve tryptone (1%), beef extract (0.3%), and sodium chloride (0.3%) in distilled water; add 0.4% agar and heat to dissolve; adjust the pH to 7.2 ± 0.2; aliquot; sterilize at 121 °C for 15 minutes; and then leave to cool and solidify. Plates were incubated at 37 °C for 12–24 h to observe bacterial motility [37]. The semidiameters of swimming areas were measured, and the images were collected using the Gel Imaging System (Tanon, Shanghai, China).

### 2.7. Observation of Bacteria and Biofilms by Field Emission Scanning Electron Microscopy (FESEM)

To observe bacterial morphology and biofilms via FESEM, WT and drug-treated bacterial cells were first inoculated onto cell climbing slides at an OD_600_ of 0.5 and then cultured in a 37 °C incubator for 24 h to allow biofilms to attach to the slide surfaces and grow sufficiently. After the specified incubation period, planktonic bacteria in the supernatant were gently removed by pipetting. The slides were then fixed with 2.5% glutaraldehyde at 4 °C for 4 h, followed by subsequent dehydration, gold sputtering, and FESEM observation (as described below). For the observation of bacterial morphology, WT and drug-treated bacterial cells were inoculated into fresh LB medium to a final OD_600_ of 0.5. The cells were cultured at 37 °C with shaking at 250 rpm for 24 h, then centrifuged to collect the pellets, which were fixed with 2.5% glutaraldehyde at 4 °C for 4 h. All samples were washed twice with phosphate-buffered saline (PBS), followed by ethanol gradient dehydration (30%, 50%, 70%, 90%, and 100%). Then, ethanol was replaced with isoamyl acetate for 2 h, followed by vacuum drying. Dried samples were mounted on conductive tape, sputter-coated with gold, and observed using FESEM (HITACHI, SU8600, Tokyo, Japan) to analyze bacterial and biofilm structures and morphology [33].

### 2.8. Determination of Cell Membrane Integrity

Cell membrane integrity of 14028 strain was evaluated using confocal laser scanning microscopy (CLSM) (Leica, TCS SP8 SR, Wetzlar, Germany): Bacteria (the initial inoculation concentration was OD_600_ of 0.5) were treated with 0MIC (WT), 1/2MIC, and MIC BH for 12 h, then centrifuged at 5000× *g* [38]. Pellets were washed three times with PBS and resuspended in PBS. Bacterial suspensions were mixed with 2.5 μM SYTO9 and 5 μM PI, incubated at 30 °C for 25 min, and washed with PBS before CLSM imaging. Fluorescence was detected at excitation/emission wavelengths of 488/520 nm (SYTO9) and 535/617 nm (PI). Since PI (red fluorescence) only penetrates cells with damaged membranes, while SYTO9 (green fluorescence) stains all cells, membrane integrity was evaluated by the ratio of red-to-green fluorescence signals [34]. The same method was used to determine membrane integrity after bacterial infection of host cells.

### 2.9. Reverse Transcription Quantitative Real-Time PCR (RT-qPCR)

Bacterial cultures were diluted in LB medium to an OD_600_ of 0.5, and 14028 strain was treated with BH at concentrations ranging from 1/8MIC to MIC. Cultures were incubated at 37 °C for 12 h, with untreated WT bacteria serving as the control. Bacterial sediments were collected by centrifugation, and total RNA was extracted using a MolPure^®^ Bacterial RNA Kit (Yeasen, Shanghai, China). Complementary DNA (cDNA) was synthesized via reverse transcription using the EasyScript^®^ One-Step gDNA Removal and cDNA Synthesis SuperMix (TransGen Biotech, Beijing, China), followed by quantitative PCR (qPCR) of type III/VI secretion system-related genes using the PerfectStart^®^ Green qPCR SuperMix (TransGen Biotech, Beijing, China). For quantification of autophagy-related genes, cells were treated with BH 2 h post-infection and harvested 12 h post-infection. And total RNA was extracted using the RNAeasy^™^ Animal RNA Isolation Kit with Spin Column (Beyotime, Shanghai, China). The subsequent operation was consistent with the bacterial procedure. *RpoD* (for bacteria) or *actin* (used in cells) gene was used for normalization [39,40]. The relative transcription levels of genes were analyzed using the 2^−ΔΔCT^ method. Primer sequences for all qPCR reactions are provided in Table 1.

### 2.10. Determination of Cytotoxicity and Intracellular Bacterial Load

For cytotoxicity testing, we first examined whether BH was toxic to cells. Raw264.7 cells were treated with BH at concentrations from 1/4MIC to 8MIC for 6 h, with untreated cells as the control, and cytotoxicity was assessed using the LDH Cytotoxicity Assay Kit (Beyotime, Shanghai, China) [41]. Next, we examined the therapeutic effect of BH on cells after bacterial infection. Bacterial cultures were diluted in LB medium to an OD_600_ of 0.5, and WT bacteria were used as a negative control; cells were infected with bacteria at an MOI of 100, and uninfected cells were used as a positive control. Two hours after bacterial infection, un-bound bacteria (bacteria that had not entered the cells) were washed away with PBS. The medium was then replaced with fresh medium, and the cells were treated with BH. After an additional 6 h, the cytotoxicity was detected using the MTT assay (purchased from Beyotime, Shanghai, China). For intracellular bacterial load quantification, drug-treated cells were treated with 0.1% Triton X-100 at 37 °C for 10 min to lyse these cells. The lysates were serially diluted, spread onto LB agar plates, and incubated for 12–24 h. CFUs were counted to calculate the bacterial load within the cells.

### 2.11. Enzyme-Linked Immunosorbent Assay (ELISA)

Fresh 14028 strain cultures were diluted in LB medium to an OD600 of 0.5, and the WT strain was used as a positive control. Raw264.7 cells were infected with bacteria at an MOI of 100, and uninfected cells were used as a negative control. Two hours after bacterial infection, bacteria that had not entered the cells were washed away with PBS. The medium was then replaced with fresh medium, and the cells were treated with BH. After an additional 6 h, the cell supernatant was collected, and the concentrations of IFN-β, TNF-α, and IL-6 were measured using an ELISA kit (Beyotime, Shanghai, China) according to the manufacturer’s instructions.

### 2.12. Determination of Reactive Oxygen Species (ROS)-Related Indicators

Cells were harvested 6 h post-infection, following the same cell treatment protocol as described for the ELISA. Activities of superoxide dismutase (SOD), peroxidase (POD), and catalase (CAT), as well as the content of malondialdehyde (MDA), were assayed using corresponding detection kits (Solarbio, Beijing, China) according to the manufacturer’s instructions.

### 2.13. CLSM for Autophagy Detection

Cells were processed 12 h post-infection, following the same cell treatment protocol as described for the ELISA. Autophagic structures were stained using an Autophagy Detection Kit (MDC Staining, Beyotime) according to the manufacturer instructions. Then, the cells were visualized using CLSM, and monodansylcadaverine (MDC) fluorescence was detected at excitation/emission wavelengths of 488/520 nm.

### 2.14. Transmission Electron Microscopy (TEM) for Autophagosome Detection

Cells were harvested 12 h post-infection and processed for TEM (HITACHI, HT7800, Tokyo, Japan) analysis, following the same cell treatment protocol as described for the ELISA [42]. Firstly, the cells were fixed with 2.5% glutaraldehyde at room temperature for 4 h, washed three times with 0.1 M PBS, then fixed with 1% osmium tetroxide (OsO_4_) at 4 °C for another 4 h, and washed three times with 0.1 M PBS. Then, the cells were dehydrated with a gradient series of 30%, 50%, 70%, 80%, 90%, and 100% ethanol solutions, each concentration applied twice for 8 minutes to ensure complete dehydration. Next, the cells were embedded in gradient concentrations of 25%, 50%, 75%, and 100% white gel, with each concentration applied for 2 h, 8 h, 12 h, and 24 h, respectively, to promote gradual white gel penetration. Embedded samples were cured in a 55 °C oven for 48 h, then trimmed and prepared into ultra-thin slices. Sections were stained with 2% uranyl acetate and with lead citrate before visualization under a TEM to observe autophagosomes.

### 2.15. Mouse Experiments

Fresh bacterial cultures were diluted in LB medium to an OD_600_ of 0.5, treated with different concentrations of BH (with WT bacteria as the control), and incubated at 37 °C with shaking at 250 rpm for 12 h; 18 C57BL/6 female mice (20 ± 1 g/mice) aged 6–8 weeks were fasted and deprived of water for 6–12 h prior to infection, followed by oral gavage with 1 × 10^8^ CFU bacterial suspension. For the survival assay, infected mice were maintained in a clean environment at 24 °C under a 12 h light/dark cycle, with daily survival status documented. In order to quantify the bacterial load of organs, mice were divided into a PBS control group (6 mice), a WT strain infection group (6 mice), and a drug treatment group (6 mice) (infected with 1 × 10^9^ CFU via oral gavage and administered 15 mg/kg BH via intraperitoneal injection 2 h post-infection) [43]. After 72 h of infection, mice were euthanized, and spleen, liver, and intestinal tissues were collected, weighed, homogenized in 0.9% NaCl, serially diluted, and plated on LB agar for 36 h of incubation to count CFU [44]. For tissue gene expression analysis, homogenized tissues were subjected to RNA extraction, reverse transcription and quantitative real-time PCR detection using the methods described previously. For the preparation of pathological sections, the intestinal tissues were fixed in a pathological fixative solution and sent to Wuhan Saiweier Biotechnology Co., Ltd. (Wuhan, China) for pathological examination.

### 2.16. Identification of Targets for the Interaction Between 14028 Strain and BH

To investigate the binding sites of BH on 14028 strain, a GraphBAN machine learning model was employed, which integrates compound and protein features and uses a graph-based framework for interaction prediction [45]. The SMILES of BH were encoded using a graph convolutional network (GCN) layer and ChemBERTa, while 5371 protein sequences of 14028 strain (strain 14028/SGSC 2262) from the Uniprot (https://www.uniprot.org/uniprotkb?query=14028%2FSGSC+2262 (accessed on 5 May 2025)) database were encoded using a 1D convolutional neural network (CNN) and the evolutionary scale model (ESM). According to the model scheme, five experimental compound-protein interaction (CPI) datasets (BindingDB, BioSNAP, KIBA, PDBbind 2016 and C57BL/6) were used as training and validation sets at an 8:2 ratio. After model training, interactions between BH and the complete proteome of 14028 strain were predicted, and 81 proteins with a predicted_value > 0.9 were selected as candidate targets (Supplementary Table S1).

### 2.17. Bioinformatics Combined with Molecular Docking

The DAVID database (https://davidbioinformatics.nih.gov/ (accessed on 6 May 2025)) was used to perform GO/KEGG enrichment analysis on candidate genes, and significantly enriched GO terms and KEGG pathways were screened with a threshold of corrected *p* value (e.g., FDR) < 0.05. A protein–protein interaction (PPI) network was constructed using Search Tool for the Retrieval of Interacting Genes (STRING https://cn.string-db.org/ (accessed on 6 May 2025)) with a set interaction confidence threshold > 0.7, and secretion system-related core modules were screened and visualized via Cytoscape (https://cytoscape.org/) [46]. Sequences of T3SS proteins SipB (ACY89898 https://www.kegg.jp/entry/T01714:STM14_3484 (accessed on 6 May 2025)), SseA (ACY89898 https://www.kegg.jp/entry/T01714:STM14_3106 (accessed on 6 May 2025)), and SsrB (ACY88164 https://www.kegg.jp/entry/T01714:STM14_1686 (accessed on 6 May 2025)) from NCBI were subjected to three-dimensional structure prediction by AlphaFold2 (https://alphafold.com/ (accessed on 6 May 2025)) and optimized using SWISS-MODEL (https://swissmodel.expasy.org/ (accessed on 6 May 2025)), while the molecular structure of BH (PubChem CID:6336590 https://pubchem.ncbi.nlm.nih.gov/ ), derived from its SMILES string, was optimized to its minimum energy conformation via Chem3D. Target proteins were prepared in AutoDock (https://autodock.scripps.edu/ (accessed on 6 May 2025)) Tools by removing water molecules, adding hydrogen atoms, and defining binding pockets; BH retained flexible dihedral angles for semi-flexible docking using AutoDock Vina with a search space of 16 × 16 × 16 Å, a grid spacing of 0.375 Å, and an exhaustiveness value of 25. Optimal conformations were selected based on a binding energy (ΔG) < −6 kcal/mol, and key amino acid sites were visualized and analyzed using PyMOL (https://pymol.org/ (accessed on 6 May 2025)).

### 2.18. Statistical Analysis

All experiments were performed in triplicate. Statistical analyses were conducted using SPSS (version 8.0 for Windows), and statistical significance was evaluated to determine differences between groups.

## 3. Results

### 3.1. BH Inhibits the Survival of Salmonella Typhimurium

**Figure 1:**
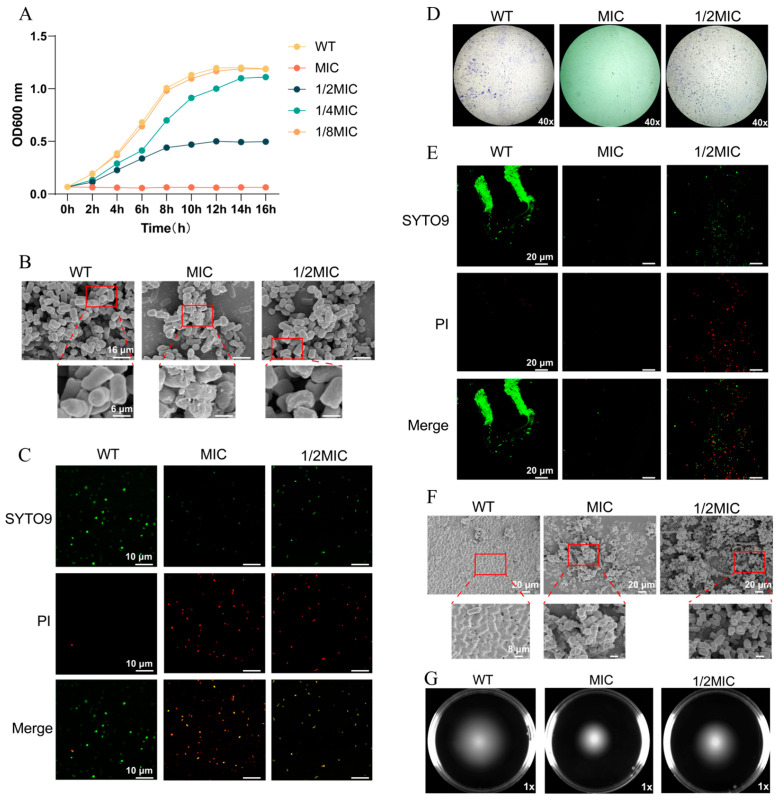
BH inhibits the survival of 14028 strain. (**A**) Growth curves of WT 14028 strain and 14028 strain treated with varying concentrations of the drug. (**B**) Morphological observations of the bacterial strains via FESEM. (**C**) Bacterial cell viability observed via CLSM. Fluorescence was detected at excitation/emission wavelengths of 488/520 nm (SYTO9) and 535/617 nm (PI). (**D**) Biofilm formation visualized by crystal violet staining. (**E**) Bacterial biofilms observed via CLSM. (**F**) Bacterial biofilm formation visualized via FESEM. (**G**) Swimming motility of bacteria after treatment with BH.

We determined the MIC and MBC of BH against *Salmonella* Typhimurium 14028 to be 125 μg/mL and 250 μg/mL, respectively. As shown in Figure 1A, BH exhibited significant antibacterial activity: MIC and 1/2MICs inhibited bacterial growth, while 1/4MIC delayed the onset of the exponential growth phase and reduced the growth rate. FESEM (Figure 1B) revealed that the MIC and 1/2MIC treatments resulted in reduced bacterial density, cell rupture, and morphological distortion. From the locally magnified images, it can be observed that the WT bacteria exhibited regular morphology and intact cell membranes without damage. In contrast, the bacteria in the MIC-treated group showed severe morphological destruction and a significant reduction in quantity. For the 1/2MIC-treated group, the bacterial morphology was also significantly damaged compared with the WT group; however, both the bacterial quantity and cell membrane integrity were better than those in the MIC-treated group (Figure 1B). Syto9/PI staining showed that, with the increasing BH concentration, red fluorescence (membrane damage) increased and green fluorescence (intact membranes) decreased. The MIC-treated group exhibited extensive red fluorescence and severe membrane disruption, while the WT bacteria predominantly showed green fluorescence with good membrane integrity (Figure 1C). Further analyses showed that BH inhibited biofilm formation (verified by crystal violet staining, Figure 1D), especially under MIC treatment, the amount of biofilm formation exhibited a marked reduction. Syto9/PI staining and FESEM (Figure 1E,F) collectively demonstrated that the degree of cell membrane damage intensified with increasing drug concentration: WT bacteria showed intense green fluorescence, indicative of intact membranes; in the MIC group, the red/green fluorescence was the weakest. This is because the biofilm was severely damaged—specifically, BH at the MIC concentration significantly reduced the formation of biofilm and led to the loss of a large number of dead bacteria. In contrast, the 1/2MIC group showed that biofilm integrity was between the two groups mentioned above (Figure 1E). Similarly, FESEM revealed that WT bacteria formed a dense biofilm, whereas the 1/2MIC group exhibited a porous and loosely structured biofilm. The biomass of biofilm in the MIC group was reduced more significantly, and the morphology of the biofilm was also severely damaged (Figure 1F). The bacterial swimming assay also exhibited the same trend (Figure 1G): upon drug treatment, the diameter of the bacterial swimming zone decreased significantly. Collectively, these results demonstrate that BH inhibits the survival of *S.* Typhimurium.

### 3.2. BH Inhibits the Expression of T3SS-Related Genes in 14028 Strain

**Figure 2:**
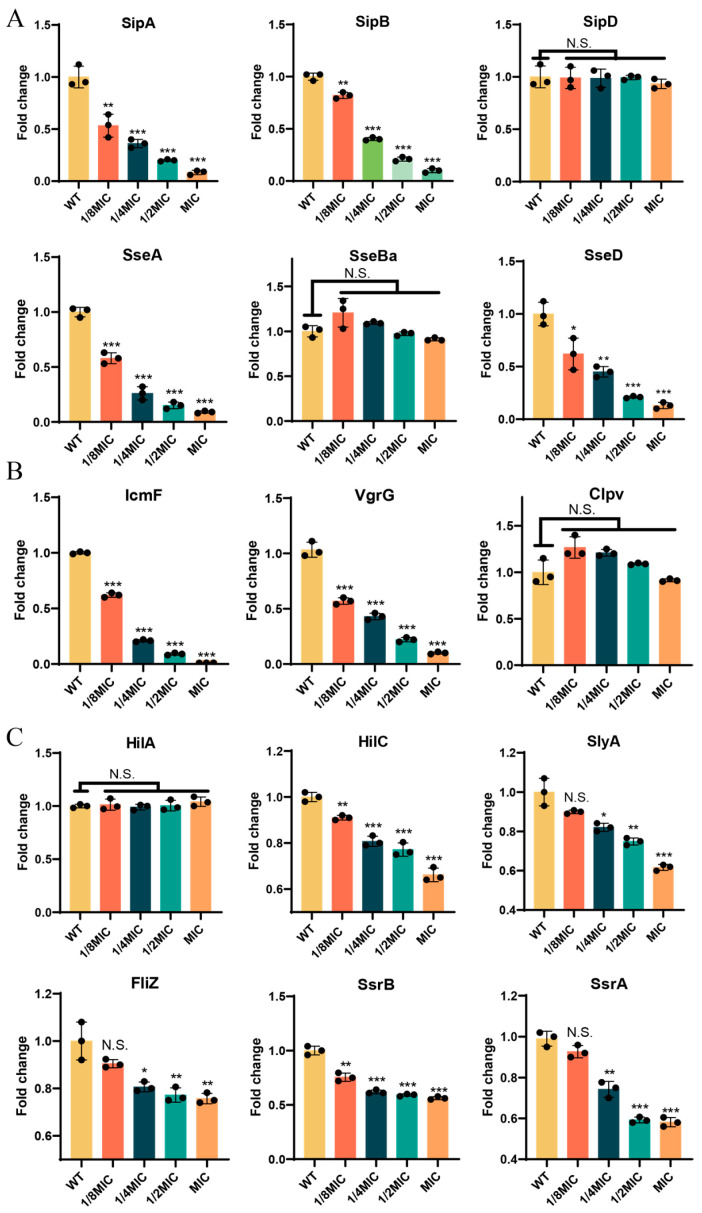
The qPCR of T3SS-related gene expression in 14028 strain. (**A**) Expression analysis of T3SS-related genes in SPI-1 (*SipA*, *SipB*, *SipD*) and SPI-2 (*SseA*, *SseBa*, SseD). (**B**) Expression analysis of T3SS-related genes. (**C**) Expression of regulatory transcription factors governing T3SS genes. Bacterial qPCR data were normalized to the *RpoD* housekeeping gene. Data are represented as mean ± SD. *n* = 3–6. N.S. represents *p* > 0.05, * *p* < 0.05, ** *p* < 0.01, *** *p* < 0.001.

Next, we investigated whether BH affected the virulence of 14028 strain [47,48]. To demonstrate this, we treated bacteria with BH and detected the expression of T3SS and the type VI secretion system (T6SS)-related genes. The reason for detecting the expression of T3SS- and T6SS-related genes is that both secretion systems are key determinants of *S.* Typhimurium virulence—the former mediates cell invasion and intracellular survival, while the latter participates in intestinal microbiota competition and immune regulation [49,50,51]. The T3SS of *S.* Typhimurium is divided into two pathogenicity islands: *Salmonella* Pathogenicity Island 1 (SPI-1) and *Salmonella* Pathogenicity Island 2 (SPI-2) [52,53]. As shown in Figure 2A, SPI-1-encoded genes *SipA* and *SipB* were significantly downregulated (*p* < 0.01) by BH: *SipA* expression decreased by 47% at 1/8MIC and was almost not expressed at MIC, while *SipB* showed a 90% reduction at MIC. Conversely, *SipD* remained unaffected (*p* > 0.05). Similarly, SPI-2-encoded genes *SseA* and *SseD* were downregulated (*p* < 0.05), while *SseBa* remained unchanged (*p* > 0.05). In the T6SS (Figure 2B), BH showed significant inhibitory effects (*p* < 0.001) on *IcmF* and *VgrG*, while *ClpV* did not (*p* > 0.05). These data suggest that *SipD*, *SseBa*, and *ClpV* may not participate in the BH-mediated response. We further examined the transcription factor expression of T3SS (Figure 2C), in which *HilA* and *HilD* are transcription factors regulating SPI-1, *SlyA* is a global regulatory transcription factor, *FilZ* is a transcription factor regulating flagella, and *SsrA*/*SsrB* is a two-component system regulating SPI-2. Consistent with our hypothesis, most transcription factors were significantly inhibited (*p* < 0.01) at 1/2MIC and MIC. Overall, these results indicate that BH inhibits the expression of T3SS-related genes in 14028 strain.

### 3.3. BH Treatment Can Enhance the Viability of 14028 Strain-Infected Cells

**Figure 3:**
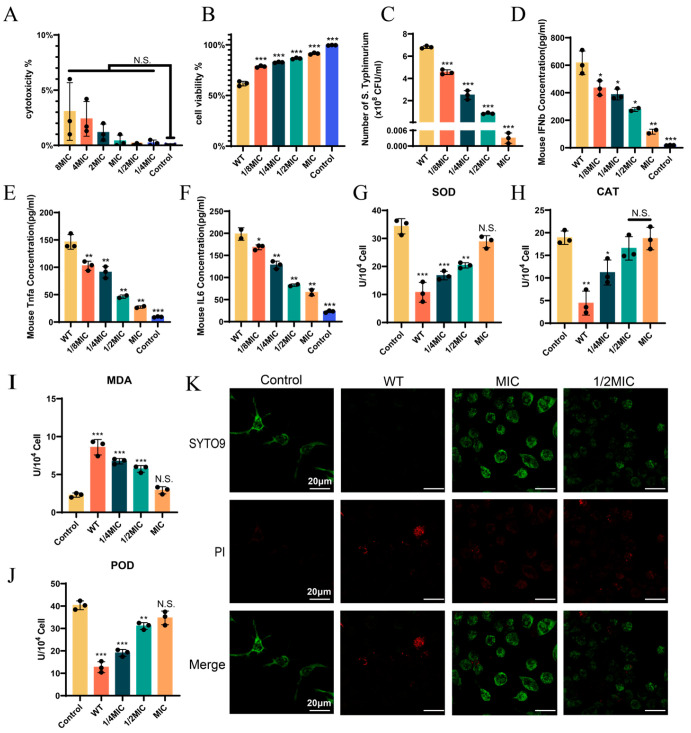
BH treatment can enhance the viability of 14028 strain-infected cells. (**A**) Detection of BH-induced cytotoxicity. (**B**) The viability of cells after drug treatment. (**C**) Quantification of intracellular bacterial load after drug treatment. (**D**–**F**) The protein levels of IFN-β (**D**), TNF-α (**E**), and IL-6 (**F**) secreted by cells treated with drugs were quantified. (**G**–**J**) Enzymatic activities of antioxidant enzymes SOD (**G**), CAT (**H**), and POD (**J**), and the oxidative stress marker MDA (**I**) were measured in cells treated with drugs. (**K**) The viability of cells after drug treatment was visualized by CLSM. Data are represented as mean ± SD. *n* = 3–6. N.S. represents *p* > 0.05, * *p* < 0.05, ** *p* < 0.01, *** *p* < 0.001.

We first evaluated whether BH had potential toxicity to Raw264.7 cells. As shown in Figure 3A, even at 8MIC, the cytotoxicity remained at 3.27%, indicating that BH did not cause significant (*p* > 0.05) cell damage. Next, we administered drug treatment to cells after they were infected with bacteria. We found that the cytotoxicity in cells infected with the 14028 strain alone was 38.3%. With increasing drug concentration, cell viability improved significantly (*p* < 0.001). At the MIC, cell viability reached 91.3% (Figure 3B). Intracellular bacterial load quantification demonstrated that as the concentration of BH increased, the bacterial load gradually decreased (Figure 3C). BH effectively inhibited the intracellular survival of *Salmonella*. ELISA (Figure 3D–F) further showed dose-dependent reductions in the release of key inflammatory mediators: interferon-β (IFN-β), tumor necrosis factor-α (TNF-α), and interleukin-6 (IL-6), all of which were significantly different (all *p* < 0.05) from those in WT infection. This finding reflects that BH attenuated the host inflammatory response. We also assessed reactive oxygen species (ROS)-related parameters (Figure 3G–J). The activities of antioxidant enzymes SOD, CAT, and POD were restored during drug treatment: at 1/4MIC, their activities rebounded to over 47%, and at MIC, all the enzyme activities recovered to over 84% (*p* > 0.05) [54]. For MDA, a key oxidative stress marker, BH treatment reduced lipid peroxidation in a concentration-dependent manner, with MDA levels returning to the uninfected control levels at MIC (*p* > 0.05) [55]. CLSM (Figure 3K) showed that compared with the WT group, cells co-treated with the drug and bacteria displayed an increase in green fluorescence (indicative of viable cells) and a corresponding decrease in red fluorescence (indicative of damaged cells). In summary, BH treatment can enhance the viability of 14028 strain-infected cells.

### 3.4. BH Enhances Autophagy in Macrophages via Inhibition of T3SS

**Figure 4:**
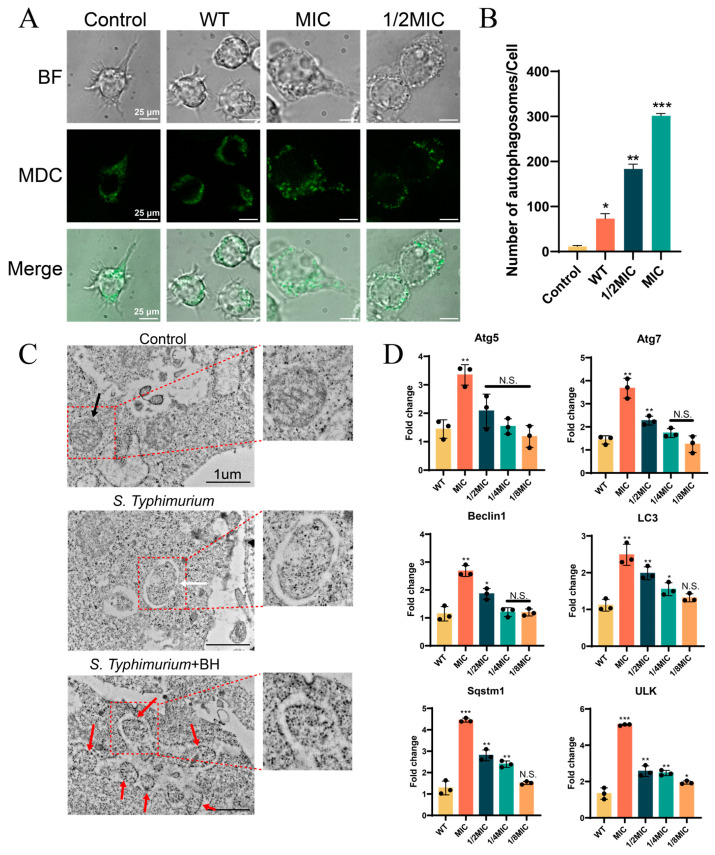
BH enhances autophagy in macrophages via inhibition of T3SS. (**A**) CLSM was used to observe autophagy in Raw264.7 cells infected with bacteria treated with or without BH. (**B**) The quantification of intracellular autophagosomes in (**A**). (**C**) TEM was used to observe autophagy in Raw264.7 cells. The drug concentration used was the MIC. The black arrow indicates normal mitochondria; the white arrow indicates mitochondrial autophagosomes, and the red arrows indicate increased autophagosomes. (**D**) qPCR was performed to detect autophagic genes in Raw264.7 cells. Cellular qPCR data were normalized to the Actin housekeeping gene. Data are represented as mean ± SD. *n* = 3–6. N.S. represents *p* > 0.05, * *p* < 0.05, ** *p* < 0.01, *** *p* < 0.001.

In previous studies, BH downregulated the expression of T3SS-related genes (Figure 2). According to existing literature, *S.* Typhimurium can inhibit host autophagy via the T3SS at the late stage of infection, thereby promoting its intracellular survival [7]. Thus, we hypothesized that BH enhanced macrophage autophagy by inhibiting T3SS, thereby reducing intracellular bacterial survival. We have confirmed in Figure 3C that BH does indeed affect the intracellular survival of *Salmonella*. To validate the effect of BH on cellular autophagy, we assessed autophagy in Raw264.7 cells infected with 14028 strain (Figure 4A), using MDC to specifically label autophagosomes through ion trapping and selective binding to membrane lipids. Figure 4B shows the quantification of intracellular autophagosomes. Compared to normal cells, the number of autophagosomes (green puncta) in the WT bacteria-infected groups was significantly lower than that in the drug-treated bacteria-infected groups (*p* < 0.01). In addition, the number of autophagosomes increased with increasing drug concentration (Figure 4A,B). TEM observations were consistent with confocal microscopy results (Figure 4C), and it was observed that the mitochondrial structure of normal cells (Control) was normal. After infection with *Salmonella*, the internal structure of mitochondria was significantly altered, accompanied by cristae loss and the formation of mitophagosomes. After treatment with BH, a sharp increase in the number of autophagosomes was observed. Quantitative PCR further confirmed significant upregulation (at MIC, *p* < 0.01) of autophagy-related genes (*Atg5*, *Lc3*) following BH treatment [56,57]. Compared with the control group, *Atg5* and *Lc3* were upregulated 3.3-fold and 2.2-fold, respectively, at MIC, while other genes showed at least a 2.6-fold upregulation (Figure 4D). Collectively, these findings demonstrate that BH enhances macrophage autophagy via inhibition of T3SS, thereby reducing *S.* Typhimurium intracellular survival.

### 3.5. BH Reduced S. Typhimurium-Induced Gastroenteritis Symptoms in Mice

**Figure 5:**
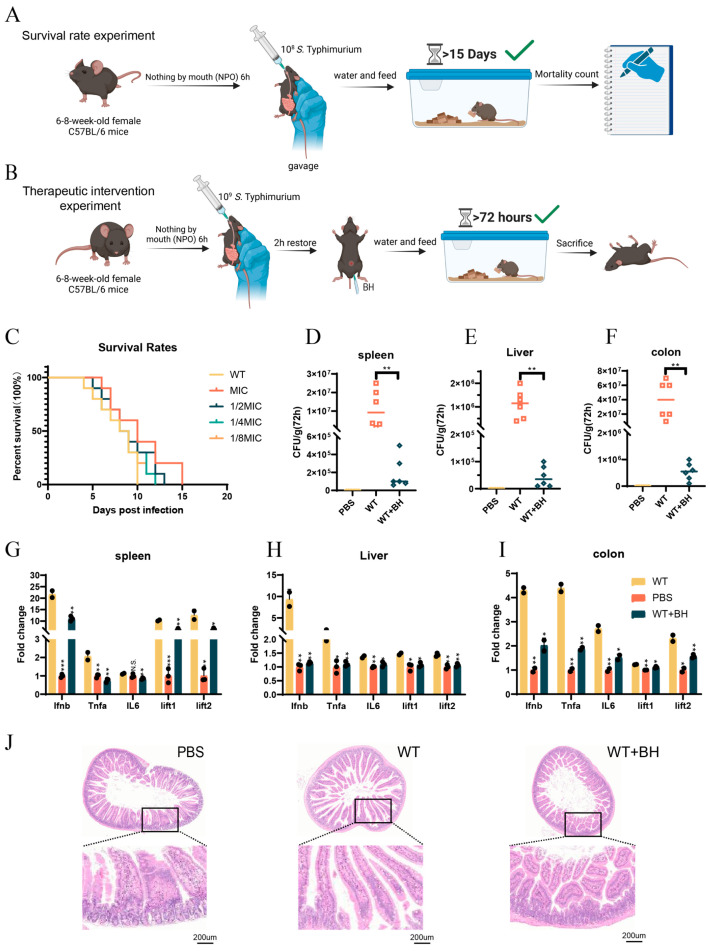
BH reduced *S.* Typhimurium-induced gastroenteritis symptoms in mice. (**A**,**B**) A survival model of mice infected with bacteria (**A**) and an experimental model of mice treated with BH (**B**). (**C**) Survival rates in mice with or without BH treatment. (**D**–**F**) The bacterial loads in mouse spleen (**D**), liver (**E**), and colon (**F**) tissues were quantified. (**G**–**I**) Interferon and inflammatory cytokine levels in the spleen (**G**), liver (**H**), and colon (**I**) of mice were assessed. (**J**) Hematoxylin and eosin (HE)-stained small intestine sections were obtained from mice treated with PBS, infected with bacteria, or drug-treated mice. Data are represented as mean ± SD. *n* = 3–6. N.S. represents *p* > 0.05, * *p* < 0.05, ** *p* < 0.01, *** *p* < 0.001.

After confirming that BH can inhibit the intracellular survival of *S.* Typhimurium through autophagy, we further investigated whether BH has a regulatory effect on *S.* Typhimurium-induced murine gastroenteritis model. We first established a survival model of mice infected with bacteria and an experimental model of mice treated with BH (Figure 5A,B), followed by comprehensive analyses of survival curve and pathological parameters. Survival curve analysis showed that mice infected with WT bacteria all died by day 10 ± 0.1, whereas the MIC-treated group exhibited a 5 ± 0.5-day survival extension, and the 1/2MIC, 1/4MIC, and 1/8MIC groups survived 3 ± 0.1 days, 2 ± 0.3 days, and 2 ± 0.6 days longer, respectively (survival curve analysis, Figure 5C), confirming that BH significantly prolonged the survival time of infected mice. Subsequently, bacterial loads in mouse tissues were assessed (Figure 5D–F). Following drug treatment, bacterial loads in the spleen, liver, and colon were significantly reduced (*p* < 0.01) compared to the WT group. Quantitative experiments based on mouse tissues (Figure 5G–I) showed that compared with the WT group, drug treatment reduced (*p* < 0.05) the expression levels of interferons and inflammation-related genes in mouse tissues. All tested genes showed a trend towards reduced inflammation after treatment. HE staining of the small intestine (Figure 5J) further showed that BH treatment alleviated WT-induced small intestinal crypt atrophy, indicating improved tissue integrity. Collectively, these findings demonstrate that BH improves gastroenteritis in mice by reducing bacterial virulence and inhibiting an excessive inflammatory response.

### 3.6. Explore the Interacting Proteins of BH Using the GraphBAN Model

**Figure 6:**
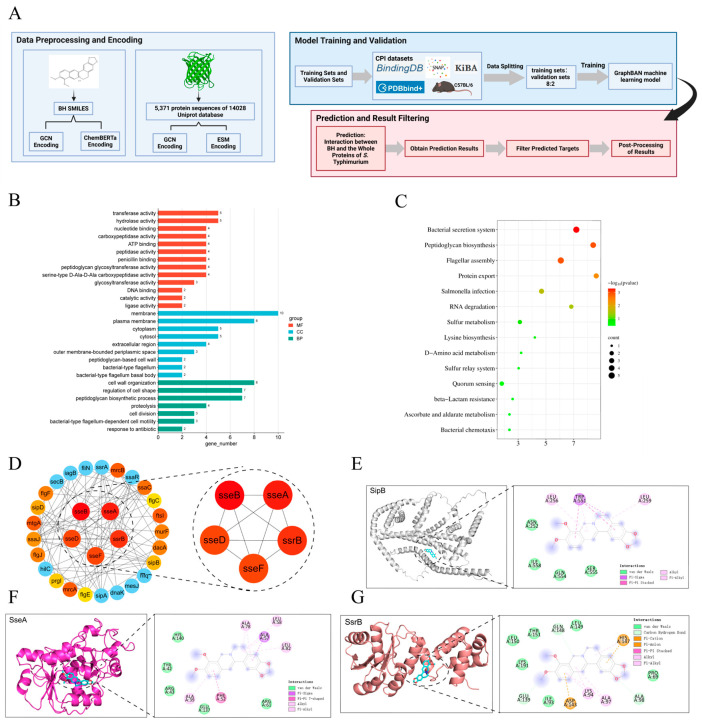
Explore the interacting proteins of BH using the GraphBAN model. (**A**) The prediction process of GraphBAN model. (**B**) GO enrichment analysis was performed on differentially expressed genes (DEGs) to identify the most significantly enriched pathways at a threshold of Padjust ≤ 0.05. (**C**) Scatter plot of KEGG enrichment of DEGs. (**D**) A PPI network was constructed using STRING with a confidence score > 0.7, and secretion system-related core modules were screened and visualized via Cytoscape. (**E**–**G**) Molecular docking predicted the amino acid binding sites of BH with key proteins SipB (**E**), SseA (**F**), and SsrB (**G**). A binding energy (ΔG) < −6 kcal/mol was defined as favorable binding capacity between small molecules and target proteins.

Here, we used the currently accurate machine learning model GraphBAN for predicting molecular interactions [45]. The prediction process of the GraphBAN model is shown in Figure 6A. The Gene Ontology (GO) enrichment analysis in Figure 6B reveals that the genes screened for interaction with BH mainly encoded transferases and hydrolases, which are located on the plasma membrane and involved in cell wall organization and cell morphogenesis. These findings are consistent with our prior observation that BH treatment alters bacterial cell morphology (Figure 1). KEGG pathway (Figure 6C) analysis identified significant enrichment of BH-interacting genes in bacterial secretion systems, peptidoglycan biosynthesis, and flagellar assembly, while PPI network (Figure 6D) analysis highlighted strong associations between BH-interacting proteins and the T3SS, confirming the reliability of the data in Figure 1 and Figure 2. Molecular docking identified the key amino acid sites that interact with BH on SipB (SPI-1 effector), SseA (SPI-2 effector), and SsrB (SPI-2 transcription factor) of the T3SS (Figure 6E,F). The binding free energies were −7.5 kcal/mol (SipB), −6.9 kcal/mol (SseA), and −7.2 kcal/mol (SsrB), all below the −6 kcal/mol threshold indicative of strong binding affinity. Collectively, these results demonstrate robust binding affinity between BH and key T3SS proteins, underscoring their role in mediating BH’s antibacterial activity.

## 4. Discussion

This study reveals for the first time that natural product BH reshapes the *Salmonella*-host interaction network through a dual-track mechanism of transcriptional repression and protein interaction. Molecular docking (Figure 6) showed that BH binds to key structural domains of the T3SS effector protein SipB and transcription factor SsrB. This binding affinity may suggest a potential role of BH in interfering with the interaction between effector proteins and host autophagy inhibitors (such as the Bcl-2 family) [58]. Meanwhile, low-dose BH (1/8MIC) can downregulate the expression of T3SS core genes, such as a 47% decrease in *sipA* expression, inhibiting virulence synthesis at the transcriptional level (Figure 2). This mechanism differs from existing inhibitors: quercitrin inhibits *HilA* to downregulate SPI-1, while fusaric acid derivative SL-19 blocks SipC secretion; both fail to target multiple effector regulations [59,60]. The dual-track regulation of BH resulted in a 3.2-fold and 2.2-fold upregulation of *Atg5*/*Lc3* gene expression in Raw264.7 cells (Figure 4), confirming the inhibitory effect of BH on bacterial virulence and its mediation of host autophagy clearance restoration [61].

**Figure 7:**
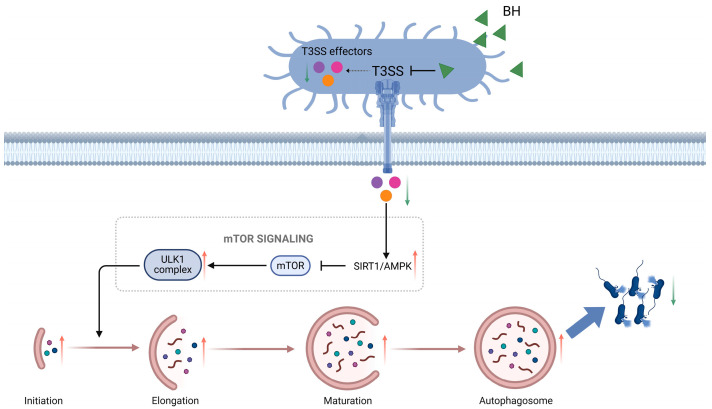
The schematic diagram summarizes the novel mechanism by which BH influences the virulence of *Salmonella* Typhimurium. Specifically, *S.* Typhimurium uses T3SS to secrete effector proteins that suppress autophagy via the SIRT1/AMPK/mTOR axis, enabling intracellular survival. Upon interacting with *S.* Typhimurium, BH inhibits the secretion of effector proteins, thereby accelerating autophagic flux and suppressing bacterial persistence and virulence within host cells.

In-depth analysis shows that BH reconstructs the host autophagy regulatory network by relieving the inhibition of the SIRT1/AMPK/mTOR pathway by T3SS. We therefore propose a novel molecular mechanism by which BH inhibits *Salmonella*-induced gastroenteritis via the T3SS (Figure 7): under normal infection conditions, *S.* Typhimurium injects effector proteins through its T3SS to suppress the expression of SIRT1/AMPK, weakening the original inhibition of mTOR and thus reducing autophagosome formation; this prevents lysosomes from degrading bacteria, allowing the bacteria to survive intracellularly. However, upon drug treatment, the drug downregulated the expression of T3SS effector proteins and transcription factors, thereby increasing the inhibition of mTOR. This led to increased autophagosome formation and a decrease in intracellular bacterial survival. It is worth noting that BH’s effect of transforming macrophages from “bacterial shelters” to “clearance actuators” significantly reduces intracellular bacterial load, providing the first structural biology evidence for drug intervention targeting pathogens through selective autophagy.

In the C57BL/6 mouse infection model, BH treatment (15 mg/kg) showed significant anti-infective effects: the median survival time was extended from 10 days to 15 days, the bacterial load in the spleen/liver was significantly reduced, and pathological damage such as intestinal crypt atrophy was effectively alleviated. Its protective effect was consistent with the drug-dependent decrease in IFN-β and TNF-α, confirming the improvement of infection outcomes through the dual pathways of “inhibiting bacterial invasion and reducing immunopathology”. More notably, BH maintained a cell survival rate of >90% at 8MIC, which is significantly safer than many similar natural products. In contrast, although curcumin can improve the survival rate of septic mice, the cell survival rate at 8MIC is only 68%, and its inhibitory effect on bacterial load is not as good as that of BH [62,63,64]. The cell survival rates of resveratrol and andrographolide at 8MIC were 75% and 62%, respectively, and there were limitations in their antibacterial spectrum or anti-inflammatory activity [65,66,67,68]. This “low toxicity and high efficiency” characteristic lays a key foundation for its clinical translation, while avoiding the contradiction of traditional antibiotics “sterilization-induced endotoxin release,” demonstrating unique advantages in the treatment of drug-resistant bacterial infections [69]. Meanwhile, this property also expands BH’s in vitro application scenarios, endowing it with clear potential in disinfection and food preservation. In in vitro applications, it not only acts on target bacteria efficiently but also avoids damaging human contact cells or food matrices. This “complementary advantages in in vivo and in vitro applications” feature not only compensates for BH’s low oral bioavailability but also provides data support for its diversified applications in drug-resistant bacteria prevention and control, as well as food hygiene and safety fields.

Although this study established a co-targeted therapeutic paradigm of “toxicity autophagy”, there are still four scientific dimensions that need to be further explored: (1) In clinical multidrug-resistant strains, the inhibitory efficiency of BH on T3SS may be reduced due to the compensatory effect of T6SS. For example, multidrug-resistant strains of *S.* Typhimurium can secrete VgrG family proteins through T6SS, partially replacing the host cell invasion function of T3SS effector proteins [70]; when the drug-resistant strain of Pseudomonas aeruginosa has impaired T3SS function due to drug inhibition, T6SS-mediated secretion of Hcp1 and VgrG2 is significantly upregulated to maintain adhesion and virulence to epithelial cells [71]. This prompts the need to verify whether BH simultaneously inhibits the T3SS/T6SS secretion system (which can be verified through double knockout strain experiments). (2) Although potential targets (SipB, SseA, SsrB) were identified, there was no experimental confirmation of these interactions (e.g., pull-down, SPR, or site-directed mutagenesis). (3) The oral bioavailability of BH (approximately 12.3%) may limit its effectiveness in treating intestinal infections, and the pharmacokinetic properties need to be optimized through nano-formulations [72,73]. (4) The specific signaling nodes in autophagy activation are not yet fully understood, and further analysis of autophagic flux signals is needed. These directions are highly in line with the current trend of “combination therapy of antiviral drugs and host-directed therapy”, providing a clear scientific path for subsequent research [74].

The core value of this study lies in breaking through the single-targeting limitations of traditional anti-virulence strategies. By elucidating the synergistic mechanism by which BH regulates the T3SS and host autophagy, a new therapeutic paradigm of “bacterial virulence inhibition-host defense enhancement” has been established for improving *Salmonella*-induced gastroenteritis. While the current findings are derived from specific *Salmonella* strains and murine models, their potential generalization to other intracellular pathogens (e.g., Shigella or Listeria) that rely on T3SS-like secretion systems or interact with host autophagy machinery merits exploration; further, given the high conservation of autophagic pathways and key virulence-associated signaling molecules between mice and humans, these results lay a foundation for investigating translatability to human clinical settings, particularly in optimizing therapeutic strategies for enteric infections caused by similar pathogenic mechanisms.

## 5. Conclusions

This study integrated in vitro experiments, in vivo animal models, and molecular approaches (such as RT-qPCR, molecular docking, and GraphBAN model prediction) to comprehensively elucidate the core mechanism of BH in preventing and controlling foodborne *Salmonella* contamination. BH targets *Salmonella* T3SS effector proteins, inhibits T3SS-related gene expression, activates host macrophage autophagy, and reconstructs the host autophagy regulatory network by relieving T3SS-mediated inhibition of the SIRT1/AMPK/mTOR pathway (Figure 7). Conclusively, the findings indicate that BH exerts its antibacterial effect by establishing a “virulence–autophagy” co-targeted therapeutic paradigm through “inhibiting bacterial virulence and enhancing host clearance capacity”, thereby ameliorating *Salmonella*-induced gastroenteritis.

## Figures and Tables

**Table 1:** Oligonucleotides used in this study.

Primers (For qPCR Only)	Sequence (5′-3′)	Size (bp)
*RpoD*-F	GTGAAATGGGCACTGTTGAACTG	23
*RpoD*-R	TTCCAGCAGATAGGTAATGGCTTC	24
*SipA*-F	TGCAAGCCATCAACGGTAGT	20
*SipA*-R	ATTGCACTGCAGTTTGCCAG	20
*SipB*-F	GCCGTTTTCTTATCGACGCC	20
*SipB*-R	CGTTGTGGCCGCTGTTTTTA	20
*SipD*-F	GCCAGGCTTGATATTTGGCG	20
*SipD*-R	ACCGTTGATCTGACGCCATT	20
*SseA*-F	ACCAAATCCGGGCTAAGGTG	20
*SseA*-R	CCGGGGCTTGAGCATTAAGT	20
*SseBa*-F	CAGCAAAATCCGTTTGCCGA	20
*SseBa*-R	CTCAGGCACCTCCTCTTTGG	20
*SseD*-F	TGTTGTCGGGTGTACTGACG	20
*SseD*-R	ATTGGGCCCCATTTTGTTGC	20
*IcmF*-F	GCTGGCGTAAAATCTTCGAG	20
*IcmF*-R	GGTAAACCACCAGTCGCAGT	20
*VgrG*-F	TGGCGGTAAACGACATATC	19
*VgrG*-R	TATTCCGCCAGAACCTCATC	20
*ClpV*-F	CCAGCGCCATTAGTGATTTTTC	22
*ClpV*-R	CGATCAACGAGGGCAGTATTTC	22
*HliA*-F	GGGCAGATGATACCCGATGG	20
*HliA*-R	AAGAGAGAAGCGGGTTGGTG	20
*HilC*-F	GGACTTGTTGCCAGGGATGA	20
*HilC-*R	GCGGGTGAGATCGCTGATAA	20
*SlyA*-F	AAGCCTCTGGAATTGACGCA	20
*SlyA*-R	GCAGGTTTGCCGCGAAATTA	20
*FliZ*-F	ACGCCTTGGCAATTACCTCA	20
*FliZ*-R	CTGGCGGTAAAGGGGGATTT	20
*SsrB*-F	ACGCTGACACGACCAATCAT	20
*SsrB*-R	CCTCATTCTTCGGGCACAGT	20
*SsrA*-F	TCCGATGAATGGCGTACTCG	20
*SsrA*-R	ATTGCCTGGTCCAGTAACGG	20
*Atg5*-F	AGCCAGGTGATGATTCACGG	20
*Atg5*-R	CTGGGTAGCTCAGATGCTCG	20
*Lc3*-F	TGACCCAGCTTAAGCGACTG	20
*Lc3*-R	AACCACATCCTAAGGCCAGC	20
*Atg7*-F	AGTGTTCAAGTGGCACACCA	20
*Atg7*-R	GCTTCTCCCATCCCTGGAAC	20
*Beclin1*-F	ACCCCATCCCTCTAGGTCAC	20
*Beclin1*-R	CTCCCCTCCCTAAGCTCCAT	20
*P62*-F	GACTGGCATTGAGGGACACA	20
*P62*-R	CCTTGCAACTGCACAACCTC	20
*Ulk1*-F	GAGACCGTTGCTGACTCCAA	20
*Ulk1*-R	TCCTAGAGAGAACAGGGGGC	20
*Atg14*-F	GCTGGAGTCTGTTCTGTGCT	20
*Atg14*-R	TTGCTGTAGGCGGTAGTTGG	20

## Data Availability

The datasets used and/or analyzed during the current study are available from the corresponding author upon reasonable request.

## Supplementary Materials

The following supporting information can be downloaded at: https://www.mdpi.com/article/10.3390/biom15111589/s1, Supplementary information is available for free, including a table (Supplementary Table S1) containing 81 screened proteins.

## Abbreviations

BH	Berberine hydrochloride
T3SS	Type III secretion system
PI	Propidium iodide
FBS	Fetal bovine serum
SPF	Specific pathogen-free
MIC	Minimum inhibitory concentration
MBC	Minimum bactericidal concentration
CLSI	Clinical and Laboratory Standards Institute
CFU	Colony-forming unit
WT	Wild-type
FESEM	Field emission scanning electron microscopy
PBS	Phosphate-buffered saline
CLSM	Confocal laser scanning microscopy
RT-qPCR	Reverse transcription quantitative real-time PCR
ELISA	Enzyme-linked immunosorbent assay
ROS	Reactive oxygen species
SOD	Superoxide dismutase
POD	Peroxidase
CAT	Catalase
MDA	Malondialdehyde
MDC	Monodansylcadaverine
TEM	Transmission electron microscopy
PPI	Protein–protein interaction
T6SS	Type VI secretion system
SPI-1	*Salmonella* Pathogenicity Island 1
SPI-2	*Salmonella* Pathogenicity Island 2
IFN-β	Interferon-β
TNF-α	Tumor necrosis factor-α
IL-6	Interleukin-6
GO	Gene Ontology
